# Complete Genome Sequence of a Pepper Yellow Leaf Curl Indonesia Virus Isolated from Tomato in Bali, Indonesia

**DOI:** 10.1128/MRA.00486-20

**Published:** 2020-06-18

**Authors:** Yutaro Neriya, Runa Izumi, Fariha Wilisiani, Sedyo Hartono, G. N. Alit Susanta Wirya, Hisashi Nishigawa, Tomohide Natsuaki

**Affiliations:** a School of Agriculture, Utsunomiya University, Utsunomiya, Tochigi, Japan; b Utsunomiya Junior College Attached High School, Utsunomiya, Tochigi, Japan; c Department of Agrotechnology, Faculty of Agriculture, STIPER Institute of Agriculture, Yogyakarta, Indonesia; d Department of Crop Protection, Faculty of Agriculture, Gadjah Mada University, Yogyakarta, Indonesia; e Department of Agroecotechnology, Faculty of Agriculture, Udayana University, Bali, Indonesia; Portland State University

## Abstract

We report a complete genome sequence of a pepper yellow leaf curl Indonesia virus (PepYLCIV) isolated in Bali, Indonesia. This virus shares around 90% identity with other PepYLCIV DNA-As and 86% identity with DNA-Bs, suggesting that it is a novel isolate of PepYLCIV.

## ANNOUNCEMENT

Most begomoviruses (family *Geminiviridae*, genus *Begomovirus*) possess bipartite circular, single-stranded DNA genomes. Begomoviruses are transmitted by whitefly and cause typical mosaic, yellowing, curling symptoms on leaves and stunted growth of plants, resulting in serious damage to crop production in tropical and subtropical regions.

Plants infected with begomoviruses were reported on Bali Island, Indonesia (1), but no complete genome sequence of a begomovirus has been deposited in the DDBJ/ENA/GenBank database.

In August 2019, we collected tomato leaves showing yellowing and a mosaic pattern, typical of begomovirus infections in Bali (8°14′8″S, 115°24′19″E). Total DNA was extracted from collected tomato leaves using a DNeasy plant minikit (Qiagen, Germany), followed by PCR amplification (BIOTAQ, Bioline, UK) of a part of the DNA-A components of the begomovirus genome, using universal primers (UPV1 and PAV1c715; product size, 1,622 nucleotides [nt]) (2). Amplified fragments were cloned into the pGEM-T Easy vector (Promega, USA) or pCRZeroT (3), and at least three independent plasmids were sequenced using the BigDye Terminator version 3.1 cycle sequencing kit and the Applied Biosystems 3500 genetic analyzer (Thermo Fisher Scientific, USA) with vector-specific primers (5′-GTAAAACGACGGCCAG-3′ and 5′-CAGGAAACAGCTATGACC-3′ for pGEM-T Easy; 5′-CGCAATTAATGTGAGTTAGC-3′ and 5′-GGGCCTCTTCGCTATTAC-3′ for pCRZeroT). Sequences were assembled using ATGC version 4.3.5 (Genetyx, Japan). We designed a primer set (5′-GAAGTCTGTATATATTATTGGCAAAG-3′ and 5′-CCCAAGGTATACAACAATGAC-3′; 1,207 nt) for inverse PCR to clone the remainder of the genome to amplify the circular DNA fragment, covering the complete nucleotide of the genome, and sequenced it in the same manner. DNA-B was amplified using pepper yellow leaf curl Indonesia virus (PepYLCIV)-specific primers (PepYLCIV_BFs, 5′-GTCCGACGTGGAATATGCAAGTG-3′; PepYLCIV_BRs, 5′-GTGCGTTCTTTGACACGTTGCTG-3′, 1,831 nt), and inverse PCR was performed with PYLCIV-Bali-invBF (5′-AAAAGAAGTGCGTTGTAAATACC-3′) and PYLCIV-Bali-invBR (5′-AAAAAAGAGTGCATTCCAGC-3′; product size, 1,124 nt). These products were used for cloning and sequencing, and PYLCIV-Bali-B-2360F (5′-GTTCAAACATTTTAGCCAACA-3′) was used for internal sequencing. Nucleotide sequence identities were calculated using GENETYX-MAC version 19 (Genetyx). No alphasatellite or betasatellite DNAs were detected using universal primers (4, 5).

DNA-A (2,743 nt; GC content, 41.6%) was predicted to have six open reading frames (ORFs) and DNA-B (2,721 nt; GC content, 39.5%) two ORFs, typical for bipartite Old World begomoviruses, using ORFfinder (https://www.ncbi.nlm.nih.gov/orffinder/). A BLASTn search (https://blast.ncbi.nlm.nih.gov/Blast.cgi) indicated that this virus shared high similarity with PepYLCIV isolates (Table 1). From a homology search analysis using the full-genome sequences of DNA-A and DNA-B, this isolate showed high sequence identity with PepYLCIV isolates (Table 1).

**TABLE 1:** Similarity analysis of PepYLCIV-[IN:Bali:Tom:19] with other PepYLCIV isolates

PepYLCIV isolate	Accession no. of:		Sequence identity (%) with:	
	DNA-A	DNA-B	DNA-A	DNA-B
Indonesia:Bogor:2000	DQ083764	NA^*a*^	90.4	NA
Indonesia:Bogor:Tomato:2000	DQ083765	NA	90.2	NA
Indonesia:2005	AB267834	AB267835	92.4	86.7
Indonesia:Tomato:2005	AB267836	AB267837	90.1	86.9
Indonesia:Ageratum:2005	AB267838	AB267839	91.3	85.4
Indonesia:Sumatra:Pepper:2012, BA_A6-1	LC051112	LC314792	91.5	86.2
Indonesia:Sumatra:Pepper:2012, BA_C1-1	LC051113	LC314793	89.9	85.9
Indonesia:Sumatra:Pepper:2012, BA_D1-1	LC051114	LC314794	89.4	86.6
Indonesia:Sumatra:Pepper:2012, BA_E2-1	LC051115	LC314795	89.5	87.3

a NA, not available; no sequence data were found in DDBJ/ENA/GenBank.

A total of 23 partial DNA-A sequences of PepYLCIV were reported (GenBank accession numbers LC381258 through LC381280). These sequences and this isolate shared 80.8 to 89.2% sequence identity, suggesting that our isolate is different from those previously reported.

According to the species demarcation criteria of the genus *Begomovirus* (6), this isolate was classified as a PepYLCIV. We propose that this isolate be named PepYLCIV-[Indonesia:Bali:Tomato:2019] (PepYLCIV-[IN:Bali:Tom:19]).

### Data availability.

The genome sequences of PepYLCIV-[IN:Bali:Tom:19] were deposited in the DDBJ/ENA/GenBank database under accession numbers LC542629 (DNA-A) and LC542630 (DNA-B).

## References

[1] Wilisiani F, Tomiyama A, Katoh H, Hartono S, Neriya Y, Nishigawa H, Natsuaki T 2019 Development of a LAMP assay with a portable device for real-time detection of begomoviruses under field conditions. J Virol Methods 265:71–76. doi:10.1016/j.jviromet.2018.10.005. 30321578

[2] Kon T, Hidayat SH, Ito K, Hase S, Takahashi H, Ikegami M 2005 Begomoviruses associated with leaf curl disease of tomato in Java, Indonesia. J Phytopathol 153:562–566. doi:10.1111/j.1439-0434.2005.01020.x.

[3] Motohashi K 2019 A novel series of high-efficiency vectors for TA cloning and blunt-end cloning of PCR products. Sci Rep 9:6417. doi:10.1038/s41598-019-42868-6. 31015513 PMC6478821

[4] Briddon RW, Bull SE, Mansoor S, Amin I, Markham PG 2002 Universal primers for the PCR-mediated amplification of DNA β. Mol Biotechnol 20:315–318. doi:10.1385/MB:20:3:315. 11936260

[5] Bull SE, Briddon RW, Markham PG 2003 Universal primers for the PCR-mediated amplification of DNA 1: a satellite-like molecule associated with begomovirus-DNA β complexes. Mol Biotechnol 23:83–86. doi:10.1385/MB:23:1:83. 12611272

[6] Brown JK, Zerbini FM, Navas-Castillo J, Moriones E, Ramos-Sobrinho R, Silva JCF, Fiallo-Olivé E, Briddon RW, Hernández-Zepeda C, Idris A, Malathi VG, Martin DP, Rivera-Bustamante R, Ueda S, Varsani A 2015 Revision of Begomovirus taxonomy based on pairwise sequence comparisons. Arch Virol 160:1593–1619. doi:10.1007/s00705-015-2398-y. 25894478

